# A cluster randomised controlled trial of an intervention to promote healthy lifestyle habits to school leavers: study rationale, design, and methods

**DOI:** 10.1186/1471-2458-14-221

**Published:** 2014-03-04

**Authors:** Fiona Gillison, Martyn Standage, Bas Verplanken

**Affiliations:** 1 Department for Health, University of Bath, Bath BA2 7AY, UK; 2 Department of Psychology, University of Bath, Bath BA2 7AY, UK

**Keywords:** Motivation, Physical activity, Diet, Health, Well-being, Habit, Communication, Self-determination theory, Adolescent, Modifiable risk factors

## Abstract

**Background:**

Physical inactivity and a poor diet predict lifestyle diseases such as diabetes, cardiovascular disease, and certain types of cancer. Marked declines in physical activity occur during late adolescence, coinciding with the point at which many young people leave school and enter the workforce and begin to take greater control over their lifestyle behaviours. The work outlined within this paper sought to test a theoretically-informed intervention aimed at supporting increased engagement in physical activity and healthy eating habits in young people at the point of transition from school to work or work-based learning. As actively engaging young people in initiatives based on health messages is challenging, we also tested the efficacy of financial incentives in promoting initial engagement with the programme.

**Methods/design:**

A three-arm cluster-randomised design was used. Participants were school pupils from Year 11 and 13 (i.e., in their final year of study), aged 16–18 years. To reduce contamination effects, the unit of randomisation was school. Participants were randomly allocated to receive (i) a 12-week behavioural support intervention consisting of six appointments, (ii) a behavioural support intervention plus incentives (totalling £40), or (iii) an information-only control group. Behavioural support was provided by fitness advisors at local leisure centres following an initial consultation with a dietician. Sessions focused on promoting habit formation through setting implementation intentions as part of an incremental goal setting process. Consistent with self-determination theory, all advisors were trained to provide guidance in an autonomy-supportive manner so that they were equipped to create a social context supportive of autonomous forms of participant motivation. The primary outcome was objectively assessed physical activity (via GT1M accelerometers). Secondary outcome measures were diet, motivation and habit strength. Data were collected at baseline, post-intervention (12 weeks) and 12 months.

**Discussion:**

Findings of this trial will provide valuable insight into the feasibility of promoting autonomous engagement in healthy physical activity and dietary habits among school leavers. The research also provides much needed data and detailed information related to the use of incentives for the initial promotion of young peoples’ behaviour change during this important transition.

**Trial registration:**

The trial is registered as Current Controlled Trials ISRCTN55839517.

## Background

Rising levels of obesity levels represent a significant public health concern [1], and are increasingly observed among children and young people in addition to adult populations [2,3]. Such rises are attributed to declines in physical activity and changes in diet (e.g., foods with higher caloric density, greater portion sizes) due to the changing social and economic environment over recent decades [4]. As such, a key focus for obesity prevention programmes has been to provide support for people to take control of their lifestyle behaviours in order to avoid or reverse obesity. Targeting preventative interventions towards young people provides a timely opportunity, as adolescents are already at greater risk of developing a number of chronic diseases in later life (e.g., coronary heart disease, type-2 diabetes) [5,6], yet their habits and lifestyle choices may be less well established and thus more malleable to change than they are in adulthood. The present paper presents the design of a theoretically-informed intervention that aims to promote healthy levels of diet and physical activity among adolescents leaving school for work.

To date, most interventions aimed at promoting healthy levels of physical activity and healthy eating with adolescents have been school-based [7]. However, it is only after they leave school that young people experience real independence in deciding how they will prioritise spending their free time and money, and have the opportunity to take control over their own health behaviours (e.g., which leisure activities to retain, whether to eat outside the home). The way in which adolescents negotiate the transition from school to work has also been found to be important in life domains other than health; for example, the personal goals that young people set and their level of commitment to them have been shown to be associated with future life opportunities and identity formation [8,9]. It is possible that goals and choices in relation to health behaviours around the school-work transition are also important for a young person’s future. Despite health behaviours such as physical activity and diet being malleable to change between child to adulthood e.g., [10-12], we could find no research that has yet explored adolescents’ goals in relation to physical activity and diet at this key transition period. The present work aimed to provide some initial insight into adolescents’ willingness to engage with health behaviours at this important point in their lives.

### Self-determination theory

Previous empirical work in applied clinical settings has demonstrated that in adopting any given behaviour, whether on a single occasion or repeatedly, a person must first be *motivated* to act [13]. How health professionals present instructions and interact with clients has a significant impact on client motivation, and consequently on whether or not these agreed actions are carried out e.g., [14-16]. A framework of motivation that has proved useful in promoting sustained health behaviours is self-determination theory (SDT) [17,18]. Within SDT it is hypothesised that people are more likely to persist in healthy behaviours if their motivation is autonomous. A number of modifiable antecedents within the social environment that can promote autonomous forms of motivation are also advanced within the tenets of SDT. Central to these is the premise that social contexts that are conducive to the satisfaction of the basic psychological needs for autonomy, competence, and relatedness, foster a person’s health and well-being. Empirical work grounded in SDT has shown that when people in positions of leadership or authority in health and exercise settings (e.g., physicians, dieticians, exercise trainers) provide a social context that is supportive of an individual’s exercise-related basic psychological needs, attendance increases [19-21]. Similarly, support for basic needs predicts closer adherence to clinician dietary advice, such as maintaining glycaemic control in patients with diabetes [15]. Strategies to promote basic needs based on this past research were therefore included within the intervention (see Standage & Ryan [22], for a discussion of intervention strategies supportive of each basic psychological need).

Despite reporting robust and significant effects, motivational interventions do not always result in positive behavioural outcomes for all participants [23]. This may be in part be due to a failure to account for the degree to which much of our behaviour is habitual, taking place somewhat automatically and bypassing conscious decision-making processes [23,24]. Habit theory specifies how it is adaptive for people to form strong automatic associations between familiar, frequently encountered environmental cues and their own behavioural responses; this reduces cognitive demand, freeing metal capacity for other tasks [25]. The non-conscious, automatic nature of habitual behaviour makes it less vulnerable to processes such as rationalisation, forgetfulness, or being replaced by competing (e.g., sedentary or less healthy) activities, as habits do not need to be deliberated or negotiated. As such, behaviours that become habituated (i.e., automated) are likely to persist. However, these same features that make habits so desirable in relation to positive, desired behaviours, are also the barriers that make old habits so difficult to break e.g., [24]. The present study will target a point of transition in young people’s lives when their environment changes, removing the cues that formerly prompted their habitual behaviour. According to habit discontinuity theory [26], the disruption of cue-response behavioural patterns at points of life transition (such as moving house, job, or leaving school) means that our behaviour can come much more closely aligned with conscious intentions, as it is freed from automatic processes [26].

### Implementation intentions

In promoting habit formation, the proposed work will make use of implementation intention theory [27,28]. Implementation intentions are specific plans regarding where, when, and how to act, and have been demonstrated to significantly increase the likelihood of goal attainment and the formation of new habits [25,28]. Habits and implementation intentions are thought to share a common mechanism; a specific cue in the environment (e.g., 6 pm) elicits a specific act (e.g., go running) in an approximately automatic fashion [29]. As such implementation intentions can serve as vehicles to ‘kick-start’ new habits, as the cue-response links which are initially formed as planned implementation intentions become the cue-response links underpinning new habits. In past work, the formation of implementation intentions has been found to predict both the adoption of healthy eating habits [28] and the development of a physical activity routine [30]. Further, the combination of implementation intentions with an intervention delivered in an autonomy-supportive social environment has been shown to result in significantly greater goal achievement than the use of implementation intentions alone [31]. Building on this work, in the present study SDT will provide a framework for the mode of delivery of health promotion instructions, and implementation intentions the mechanism by which behavioural responses to environmental cues are initiated and reinforced.

### Incentives

Past work has shown adolescents and young adults to be notoriously difficult to engage in health promotion initiatives, largely as a result of their low levels of perceived health risk. Therefore, innovative ways of communicating the relevance and importance of health behaviours are needed if we are to motivate young people towards action. One approach to attracting initial participation with this age group may be through the use of incentives (i.e., as a means of supporting attendance at the sessions aimed at developing initial volitional engagement in health behaviours). Past work has shown that incentives such as the provision of free groceries, cash payments, reduced-price healthy vending machine options, and food coupons are effective in promoting healthy eating in adults [32]. With young people, direct financial incentives have also been found to be effective in promoting both research response rates [33], and enrolment in health education counselling [34]. Incentives are particularly powerful in engaging individuals with low economic resources [35], which may suggest that they would have stronger effects in populations such as school leavers who are likely to be earning low wages. More work in this area is called for, as while empirical work has shown some promise in promoting positive outcomes, effect sizes have so far been small [32]. Further, the efficacy of incentives in supporting or “priming” behaviour change specifically in young adults, and the efficacy of incentives beyond cash payments have received little research attention.

There is further tension between the use of incentives and recommendations for the promotion of autonomous motivation from the perspective of SDT [36,37]. Without an informational component, incentives can undermine intrinsic motivation for existing activities as a person’s attention shifts to the controlling external factors, and they no longer perceive themselves to be acting for the inherent qualities of the activity (e.g., for enjoyment, pleasure, and satisfaction). In this scenario, when incentives are removed it is likely that the behaviour will also cease [38]. Nonetheless, extrinsic factors can be powerful motivators in the short-term, as many pro-social human behaviours are learned from a starting point of external prompts e.g., the adoption of societal values; [17]. Thus, it is possible that incentives could represent a motivational prompt for the adoption of a new behaviour. In order for the behaviour to persist a person would need to develop more autonomous motivation over time, so that they become decreasingly prompted by the incentive, and increasingly prompted by appreciating other personally meaningful benefits of the activity though the process of internalization; [39]. The aims of the present intervention would therefore be to use incentives to provide the initial impetus to prompt attendance at a health promotion programme, but once enrolled, focus on helping participants to develop more autonomous reasons for engagement [39]. Such an approach is justified as it is unlikely that adolescents would be purely intrinsically motivated towards fitness-oriented physical activity and healthy dietary choices. To minimise the risks of the incentives undermining autonomous motivation, consistent with previous work with young people [33,34], the incentives were made contingent on attendance at leisure centre appointments and not on performance or achievement of healthy behaviours (i.e., not matched to key target behaviours). Given recent government attention on the use of incentives for promoting health behaviours e.g., as presented in; [40], which departs from a theoretical perspective, formally testing the efficacy and outcomes of this approach in an applied setting is important.

### Present work

A theoretically informed intervention was initially designed to build on promising components of existing lifestyle interventions via the use of the systematic design process of intervention mapping; [41,42]. The intervention was refined through an initial qualitative needs assessment phase involving young people and the adults who work with them (teachers, youth workers and health professionals) to identify predictors and barriers to (i) healthy eating and physical activity, and (ii) engaging young people in healthy lifestyle programmes. Information from the needs assessment was combined with evidence of the behavioural determinants identified from a literature review, to specify a set of objectives that young people will need to achieve in order to engage with the programme. Recent work in behavioural science has led to improvements in the specification and testing of behaviour change techniques that influence theoretical and practical determinants of behaviour change [43,44]. In line with this established best practice, our intervention comprised a set of theoretically informed behaviour change strategies that aimed to support participants to achieve each identified objective.

A limitation of many past empirical studies related to behaviour change has been the lack of objective or reliable means by which to measure primary research outcomes. To overcome such a limitation, in the present research physical activity was monitored via Actigraph G1TM Units, a device that provides accurate accelerometry data pertaining to a person’s pattern of physical activity [45]. Diet was assessed by a well-validated Europe-wide instrument [46], which enables the findings of the present work to be viewed in context with prior and on-going dietary monitoring initiatives.

### Research aims and hypotheses

The primary aim of this work was to statistically test the efficacy of behavioural counselling sessions grounded in SDT in encouraging participants to set implementation goals to improve their physical activity and dietary habits. These aims are in line with a recent Cochrane report identifying clear advantages for approaches that combine both dietary and physical activity elements in combating risk factors for heart disease including obesity, as opposed to focusing on either factor alone [47]. A three-arm cluster randomized controlled trial (CRCT) was implemented to facilitate testing the added value of incentives for enrolling and retaining participants in the initiative, compared with both a stand-alone behavioural support and a control group.

### Hypotheses

1. School leavers receiving behavioural support will report increased involvement in regular physical activity and improvement in diet quality relative to participants in the control group.

2. School leavers receiving additional incentives for participation will report the most positive outcomes, relative to those receiving behavioural support alone. This will result from incentives prompting initial programme attendance, with the behavioural support sessions supporting subsequent autonomous engagement in health behaviours.

3. Greater autonomous motivation for change will predict increased physical activity and an improvement in diet quality.

4. The frequency and automaticity of physical activity and dietary patterns will be predicted by the formation and enactment of implementation intentions.

5. School leavers receiving behavioural support will develop stronger habits of physical activity and healthy food intake relative to participants in the control group.

## Methods/design

### Ethics

This research was approved by the University of Bath School for Health Research Ethics Approval Panel (ref. EP 07/08 57) and is registered with Current Controlled Trials (registration number: ISRCTN55839517).

### Design

The research followed a mixed-methods approach across two study phases. Phase 1 involved focus groups with school leavers to explore their views on: i) the perceived importance and role of diet and physical activity in their lives, ii) the types of exercise that appeal to them and their peers, iii) their general priorities at this time in terms of potential barriers to participation, and iv) what incentives would be of value to other school-leavers. The results of Phase 1 were used to inform and refine the planning of the intervention (i.e., Phase 2) prior to piloting the intervention components on a subsample of the target population.

Phase 2 was a three-arm cluster randomised control trial (CRCT), conducted in line with the CONSORT guidelines for CRCTs [48]. Participants were randomly allocated to one of three conditions; (1) behavioural support (BS), (2) behavioural support plus incentives (BSI), or (3) information only control group (C). Outcomes were measured following allocation to one of the three groups and at 3 months (i.e., immediately following the intervention) and 12 months post baseline.

### Participants

The target population was school pupils leaving school for work, vocational training or with no definite plans for their future, according to their plans during their final term at school.

### Inclusion/exclusion criteria

All pupils in their final year of school before seeking employment or work-based learning apprenticeships (i.e., aged 16–18) attending schools and colleges in two education authorities in south west England were eligible to take part.

### Recruitment

To reduce bias and potential contamination effects of pupils in the same friendship groups receiving different treatment, a block randomisation approach by school was used to allocate participants to one of the three trial arms. Participants were recruited at their school (e.g., during a registration period) prior to taking exams in their final year of study. To avoid the compromises to generalizability introduced through convenience sampling, the sampling frame was a complete list of schools within two education authorities in south west England (i.e., Bath and North East Somerset, and Wiltshire). Prior to randomisation, schools were stratified according to size, location and social-economic characteristics of the catchment area, so as to ensure a representative sample was retained within each experimental condition.

The study was presented by a research assistant in small class-sized groups (e.g., ≤30 students). A designated link-teacher was present to act as an accessible information point for potential participants following the initial research visit. Pupils interested in taking part provided their contact details, and were then invited to attend an initial appointment by the study team following the completion of their exams.

### Procedure

Participants were invited to attend an appointment at their local leisure centre several weeks after leaving school to provide baseline measures and written consent (Session 0). At this time, they were fitted with a GT1M accelerometer that was worn for one week. Session 1 took place one week later, and attendance at this point constituted enrolment in the study for the purposes of the intent-to-treat analysis (i.e., following completion of baseline measures). Session 1 was delivered by a dietician and Sessions 2–6 by a leisure centre fitness advisor (the same advisor on each occasion). Participants saw their advisor individually or in pairs, as they were encouraged to sign up for the scheme and engage in activities within their existing friendship groups. Following the final session, participants were contacted by the research team and asked to wear the GT1M accelerometer again for another one week assessment period. Follow-up data were collected 12 and 52 weeks following Session 1.

### Intervention

**Figure 1:**
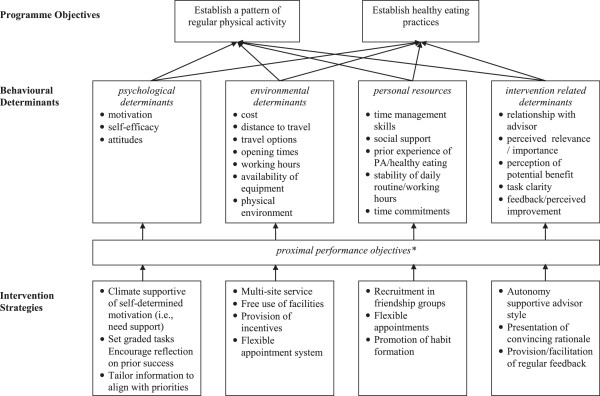
**Summary intervention map of systematic intervention design.** *Note: Full details can be provided by the authors.

The basic behavioural support intervention was designed using intervention mapping to reflect evidence-based practice in matching the determinants of behaviour that the intervention aimed to target, with specific strategies demonstrated to influence them. Core components are provided in Figure 1.

#### ***Behavioural support group (BS)***

*Session 1 (week 1)*: Participants received a 30–45 minute one-to-one session with a dietician advisor trained in the study protocol. The advisor provided tailored feedback on the participant’s current habits (referring to Actigraph data, and dietary records) as they relate to current health guidelines [49]. Discussion focused on aspects of diet and activity that the participant would consider changing, with a particular emphasis on identifying opportunities for small but significant modification that would fit within their existing lifestyle (i.e., achievable but meaningful changes). The outcome of the discussion was the selection of two specific goals for the following two week period; one relating to a change in dietary habits, and one to a change in physical activity (e.g., to eat at least one piece of fruit a day, or to swim twice a week). In line with the implementation intention approach, each goal was associated with a specific environmental cue (e.g., after breakfast, or on leaving work every Tuesday and Thursday). The goals represented the first stage in moving participants towards advised levels of physical activity and dietary guidelines.

The tone of the advice and support given followed specific practical techniques endorsed in past applied SDT research, and aimed to promote a sense of ownership/endorsement in reaching behaviour change targets. Specifically, advisors aimed to: a) support autonomy (e.g., using terms such as “you may want to, you could choose to” rather than “you should” or “you must”), b) demonstrate empathy (e.g., “I can see why you might find this difficult”), c) confirm realistic expectations, d) present a meaningful rationale as to why the activity is important, e) provide structure through ensuring that the implementation intentions are clear, simple, and linked to identifiable environmental cues (e.g., finishing work, getting up), and f) provide proximal feedback (e.g., recap on goal achievement, highlight progress since baseline).

Subsequent sessions took place with a leisure centre advisor, and focused on monitoring goal progress, and updating physical activity and dietary goals. Participants were able to access leisure facilities free of charge for the duration of the project to reduce cost barriers. At the final session (12 weeks post initiation), participants discussed their progress to date and developed a future action plan (e.g., continued gym membership, consolidation of dietary goals).

#### ***Behavioural support plus incentive group (BSI)***

Participants in the BSI group followed the same treatment protocol as the BS group, but in addition received four £10 vouchers (e.g., driving lesson, mobile phone top-up, high street shopping) spaced across the duration of the intervention. The provision of incentives was contingent on attendance at goal review appointments, not on the achievement of goals themselves.

#### ***Control group (CG)***

Participants in the control group received a one-off information session consisting of standard healthy eating and physical activity advice delivered by the research assistant. This group received no further contact until 12-week follow-up.

### Intervention delivery

A half-day training course was provided by the research team for all dieticians and leisure centre staff to familiarise them with the study protocol; namely the provision of support and information in a need supportive fashion within the tenets of SDT, and techniques for setting implementation intentions.

### Primary and secondary outcome measures

All outcome measures were recorded following allocation to the three research arms (baseline), 3- and 12-months.

#### ***Primary outcome measures***

The primary outcomes were objectively assessed physical activity (using the Actigraph GTM1 activity monitor) and diet (using the European Prospective Investigation of Cancer food frequency questionnaire [EPIC FFQ]). The Actigraph has been validated in child and adolescent populations e.g., [50] and has no external controls or display, thus preventing user-feedback influencing activity levels or the intervention.

#### ***Secondary outcome measures***

Behavioural measures were supported by the measurement of waist circumference and BMI to explore whether behavioural outcomes influenced participants’ body weight and / or composition.

Psycho-social factors that can help to explain the mechanism of adoption of new behaviours, and likely sustainability were also recorded. Specifically, motivation towards exercise was assessed using the BREQ-2 [51], basic need satisfaction (for autonomy, competence and relatedness) was measured using The Psychological Need Satisfaction in Exercise Scale [52], and habit strength for physical activity and dietary intake using the Self-Report Habit Index [53].

### Sample size

A power calculation was conducted for a three-arm CRCT based on intra-class correlations and changes in total accelerometer counts obtained from previous research [54]. For an estimated cluster size of 30 participants per centre (school), a total number of 5 clusters per condition would be necessary to provide adequate power (80%, *p* < .05) providing a total sample size estimate of 450 participants. It was estimated that drop-out would be marked for this age group, reflecting the considerable life changes at this time, geographical movement, and lack of health concern in younger age groups. Across the two districts identified for the project in the school year ending the summer prior to the start of the intervention, 1267 pupils left school for work or vocational training at 16, and a further 263 at 18; i.e., a total pool of potential participants in a one year period of 1530 young people. In order achieve the estimated sample size and accommodate high rates of attrition the intervention was offered to all school-leavers.

Trial burden for users and potential providers was addressed through user-involvement in the study design. Recruitment was staggered to retain a manageable workflow for leisure centres (although the summer months are typically relatively quiet). Participant burden in relation to potential gains was minimal; even the control group received basic health advice beyond that provided as part of usual care [55]. Attention was given to selecting parsimonious measures to reduce necessary response times.

### Statistical analysis

Differences in change over time between the intervention and control groups will be analysed using the general linear model for (a) physical activity, (b) frequency of consumption of target food groups. Using the HLM 7 software [56], cluster effects will be adjusted for by using multilevel analysis, with leisure centre representing the higher order unit of analysis. Effect sizes will be calculated to assess the degree to which any statistically significant differences are meaningful. Results will be reported using the RE-AIM (Reach, Efficacy, Adoption, Implementation and Maintenance) framework [57]. RE-AIM provides information useful to the evaluation of how generalizable the findings of a single trial may be to the general population, and reflecting how easily it can be adopted into institutional practice.

## Discussion

Within this paper, a protocol was presented which provides a number of examples of ways in which key challenges in promoting health to adolescents could be addressed during the school-to-work transition. Results from the study will provide empirical data on the efficacy of such approaches when incorporated into a systematically designed intervention. Rich qualitative data will also provide much needed and valuable information regarding the lived experiences and challenges faced during the school-to-work transition. The research presented is one of the first studies to attempt to promote the internalization of motivation for physical activity and a healthy diet, at the point when young people experience a shift in their general level of autonomy over their own lives (i.e., during the transition from school to work), thus capitalising on the discontinuity of old habits [24,25,58]. The work outlined aims to target a challenging population, given that previous studies show the lack of importance that young people attribute to health as a rationale for behaviour change [59,60]. The present intervention will also allow a test of whether aligning changes with aims to demonstrate maturity and independence are sufficient to motivate young people to adopt and pursue healthy behaviours. As few intervention studies attempt to tackle these issues, evidence such as this is overdue.

The research encompassed within this protocol will also provide an applied test of the use of incentives, to explore whether it is possible to avoid the predicted outcome of incentives undermining motivation through delivering incentives as a means of promoting initial engagement with a service, rather than rewarding the successful achievement of goals. Given government interest in the use of incentives for promoting health [40], it is timely to explore the efficacy and potentially negative unintended consequences of incentives within carefully designed empirical research.

A strength of this study is the systematic intervention design that will facilitate a process analysis to test the efficacy of strategies employed at influencing the proposed mediators of behaviour change [61,62]; namely need satisfaction, motivation, and habits. Such an approach will assist in the interpretation of findings in systematically establishing how and why the intervention did, or did not, bring about the predicted effects. This more specific information on the performance of behaviour change strategies on the mediators of change will contribute to a shared evidence base of the performance of different approaches in different populations to advance our knowledge and understanding of intervention design [43,63]. The results from this research will be reported according to Consolidated Standards of Reporting Trials (CONSORT) recommendations [64].

## Abbreviations

BMI: Body mass index; CRCT: Cluster randomized controlled trial.

## Competing interests

The authors declare they have no competing interests.

## Authors’ contributions

The study was designed by all three authors. All three authors contributed to the writing of this paper. All authors read and approved the final manuscript.

## Pre-publication history

The pre-publication history for this paper can be accessed here:

http://www.biomedcentral.com/1471-2458/14/221/prepub
